# Post-error adjustments depend causally on executive attention: Evidence from an intervention study

**DOI:** 10.3389/fpsyg.2022.1014909

**Published:** 2022-10-11

**Authors:** Qing Li, Yixuan Lin, Xiangpeng Wang, Mengke Zhang, Francis Stonier, Xu Chen, Antao Chen

**Affiliations:** ^1^Key Laboratory of Cognition and Personality of Ministry of Education, Faculty of Psychology, Southwest University, Chongqing, China; ^2^Collaborative Innovation Center for Language Ability, Jiangsu Key Laboratory of Language and Cognitive Neuroscience, School of Linguistic Sciences and Arts, Jiangsu Normal University, Xuzhou, China; ^3^Department of Curriculum and Instruction, Southwest University, Chongqing, China; ^4^School of Psychology, Shanghai University of Sport, Shanghai, China

**Keywords:** executive attention intervention, transfer, post-error accuracy, post-error adjustments, Posner cueing paradigm

## Abstract

Detecting and correcting execution errors is crucial for safe and efficient goal-directed behavior. Despite intensive investigations on error processing, the cognitive foundations of this process remain unclear. Based on the presumed relation between executive attention (EA) and error processing, we implemented a seven-day EA intervention by adopting the Posner cueing paradigm to test the potential causal link from EA to error processing in healthy adults. The experimental group (high EA, HEA) was trained on the Posner cueing paradigm, with a ratio of invalid cue (IC) trials to valid cue (VC) trials of 5:1 and a corresponding ratio of 1:1 in the active control group (low EA, LEA). We found that the EA intervention improved EA across intervention sessions. Critically, after the EA intervention, the HEA group showed that post-error accuracy (PEA) was restored to the same level as the post-correct accuracy (in comparison with the LEA group). However, post-error slowing and the flanker effect were not modulated by the EA intervention. Furthermore, we observed that the changes in the accuracy of VC trials positively predicted the changes in PEA and that the two groups were classified according to the changes in PEA with a 61.3% accuracy. Based on these results, we propose that EA causally drives error processing. And the capabilities of the “actively catch” more attention resources and the automatic mismatch processing developed after EA intervention is transferable to error processing, thereby directly resulting in the gains in post-error adjustments. Our work informs the potential cognitive mechanisms underlying this causal link.

## Introduction

Understanding the cognitive root of error processing is an important challenge in the area of error research. Effective cognitive processing triggered by errors is dependent on error monitoring and post-error attentional adjustment (Gehring et al., 1993). The former is needed to evaluate whether the actual response is a correct response, while the latter is crucial for adjusting subsequent behavior once a mismatch is detected between the actual and correct responses. Most studies on the cognitive origins of error processing have focused on the error signal itself, such as the error occurrence probability (Notebaert et al., 2009; Castellar et al., 2010), error awareness (Steinhauser and Yeung, 2010; Boldt and Yeung, 2015), and error type (Li et al., 2022). In contrast, little is known about how to explore this question from a causal perspective. For instance, intervening with regard to a key cognitive ability underlying error processing helps to improve post-error performance, which benefits both the understanding of the cognitive root of error processing and the improvement in post-error behavior. Recent studies have provided a viable basis for this pathway, showing evidence for a deficit in the later stages of error processing and the subsequent implementation of cognitive control in ADHD; this suggests that error processing is associated with attention control and regulation (Shiels and Hawk, 2010; Ehlis et al., 2018; Janssen et al., 2020). In the current study, we intended to implement an intervention program about the key cognitive ability to control and regulate attention in healthy adults, in order to extend these observations and to assess a causal link from this cognitive ability to post-error performance.

Post-error slowing (PES) and post-error accuracy (PEA) are commonly used behavioral indices that measure post-error performance. PES refers to the phenomenon that individuals slow down in the subsequent trial after an error occurs (Rabbitt, 1966). Adaptive theories and maladaptive theories are proposed according to PEA predictions. Adaptive theories hold that, after error commission, the response threshold is increased to obtain more time for goal-directed processing, resulting in increased PEA (King et al., 2010; Danielmeier et al., 2011). Nevertheless, previous studies did not find reliable correlations between PES and PEA (Ullsperger et al., 2014). Likewise, maladaptive theories propose that error commission momentarily impairs ongoing processing, resulting in decreased PEA (Notebaert et al., 2009; Houtman and Notebaert, 2013). Significantly, the recent research suggests that available attention resources play an important role in the adaptability of error processing (Li et al., 2021). This seems to indicate that attention is the core cognitive ability determining PEA.

Executive attention (EA) is a domain-general ability for voluntarily controlling attention to regulate thoughts and behavior (Posner and Digirolamo, 1998). EA is the basic component of executive function, which controls and regulates cognitive processes by utilizing attention resources. As a general ability, the processing efficiency of EA determines performance in high-level cognitive processes, such as working memory and general fluid intelligence (Engle, 2002; Kane and Engle, 2002). This characteristic of EA establishes the basis for the far-transfer effect of EA intervention. We inferred from this that an intervention on EA could effectively improve performance in high-level cognitive processes.

Although the idea of a relationship between EA and error processing can be traced back to at least the 1990s (Falkenstein et al., 1991), surprisingly, only a few studies have empirically tested this association (Murphy et al., 2006; Xiao et al., 2015; Li et al., 2021). Interestingly, all these studies implied correlations between EA and error processing. For instance, the study by Murphy et al. (2006) showed that, compared to alert conditions with sufficient attention, further error evaluation (error positivity, Pe) and remediation of these errors were impaired within sleepy conditions because of lapses in attention. In addition, Xiao et al. (2015) found that error-related negativity (ERN) amplitude in the fatigue group was smaller than that in the normal group, prompting the conclusion that sustained attention was related to error processing and that decreased attention could be a major cause of impaired error processing. Recent study also showed that when the response–stimulus interval (RSI) was long, adequate attention resources could be provided (reflecting alpha suppression), which favored adaptive post-error adjustments (Li et al., 2021). These related studies complement neuroscientific evidence indicating that EA and error processing activate a similar frontoparietal network, described as the execution control network (Ptak, 2012; Cai et al., 2015). These studies open the door to determine whether EA ability has a causal link with error processing.

An important paradigm on EA in recent decades has been the Posner cueing paradigm (Posner, 1980). In this paradigm, the target will appear in one of two locations. Before the presentation of the target, a cue indicates the possible location of the target with a certain validity. Those trials in which the cue correctly indicates the location of the target are called valid cue (VC) trials, while those in which the cue incorrectly indicates the location of the target are called invalid cue (IC) trials. This study planned to employ this paradigm as an intervention task for the following reasons. First, the Posner cueing paradigm involves IC trials that has a tendency to dominate the latent factors of attention, which provides a strong basis for the IC trials intervention to effectively improve EA (Rey-Mermet et al., 2019). Second, the IC trials need top-down attention control, whereas the VC trials reflect unconscious automatic processing, demonstrating that IC trials need EA more than VC trials (Unsworth et al., 2011; Draheim et al., 2021). As a result, if IC trial is more trained, the EA should be improved to a greater extent. Posner cueing paradigm is easy to achieve this by setting a larger number of IC trials and a smaller number of VC trials. Third, since the intervention tasks in the experimental group and the active control group need to be comparable and less disparate as possible, we adopted Posner cueing paradigm for both groups, which differed only on the ratio of IC and VC trials. This would largely exclude the influence of additional factors (e.g., stimulus attributes, response criterion, etc.) caused by different intervention tasks on the intervention results. This practice is based on the modified Posner cueing paradigm by Lin et al. (2022), which successfully improved EA. Therefore, we set up the experimental group: high EA group (HEA group, IC: VC = 5:1) and the active control group: low EA group (LEA group, IC: VC = 1:1) by manipulating the ratio of the number of IC and VC trials. In the HEA group, participants would train EA to a greater degree by shifting attention to the opposite direction of a cued stimulus at a high frequency, at which the active attention control would be executed (as most cues were predictable). In the LEA group, EA would not be trained due to the equal ratio and pseudo-random presentation between the IC and VC trials, at which passive attention control may be implemented (as the cues were unpredictable).

Consequently, the first aim of this study was to demonstrate the trainability of EA, the second aim was to test the causal link from EA to error processing, and the third aim was to clarify the underlying cognitive mechanisms to determine if there was a causal link between the two. Theoretically, we assumed that the cognitive root of error processing is EA, which would be consistent with some theories (Jentzsch and Dudschig, 2009; Li et al., 2021), and hypothesized that EA intervention improves post-error performance by effectively diminishing the attention bottleneck induced by error monitoring, and supplying more attention resources for the post-error adjustments. To measure post-error performance before and after the intervention, we employed the modified flanker task (the four-choice flanker task; Maier et al., 2008) which was more difficult than typical flanker tasks (Eriksen and Eriksen, 1974), in order to obtain adequate post-error trials for analysis. In summary, we predicted that EA would be improved during the EA intervention in the HEA group but not in the LEA group. If there was a causal link from EA to error processing, improved post-error performance should be observed only in the HEA group after intervention.

## Materials and methods

### Participants

In total, 106 healthy volunteers participated in the present study. All participants were right-handed, had normal or corrected-to-normal vision, and had no history of nervous system disease. They were randomly divided into either the HEA group (*N* = 54, 11 male, mean age = 19.59 years, *SD* = 1.49 years) or the LEA group (*N* = 52, 15 male, mean age = 19.52 years, *SD* = 1.45 years). Both groups did not differ in age (*t*(104) = 0.26, *p* = 0.073) and sex (*t*(104) = 1.01, *p* = 0.085). All participants signed informed consent prior to the experiment, and received ¥120 (~$17.7) for compensation at the end of the experiment, no matter how they performed in the experiment. The study was conducted in accordance with the principles of the Declaration of Helsinki and its later amendments and was approved by the local Human Ethics Committee for Human Research.

### Study design

**Figure 1:**
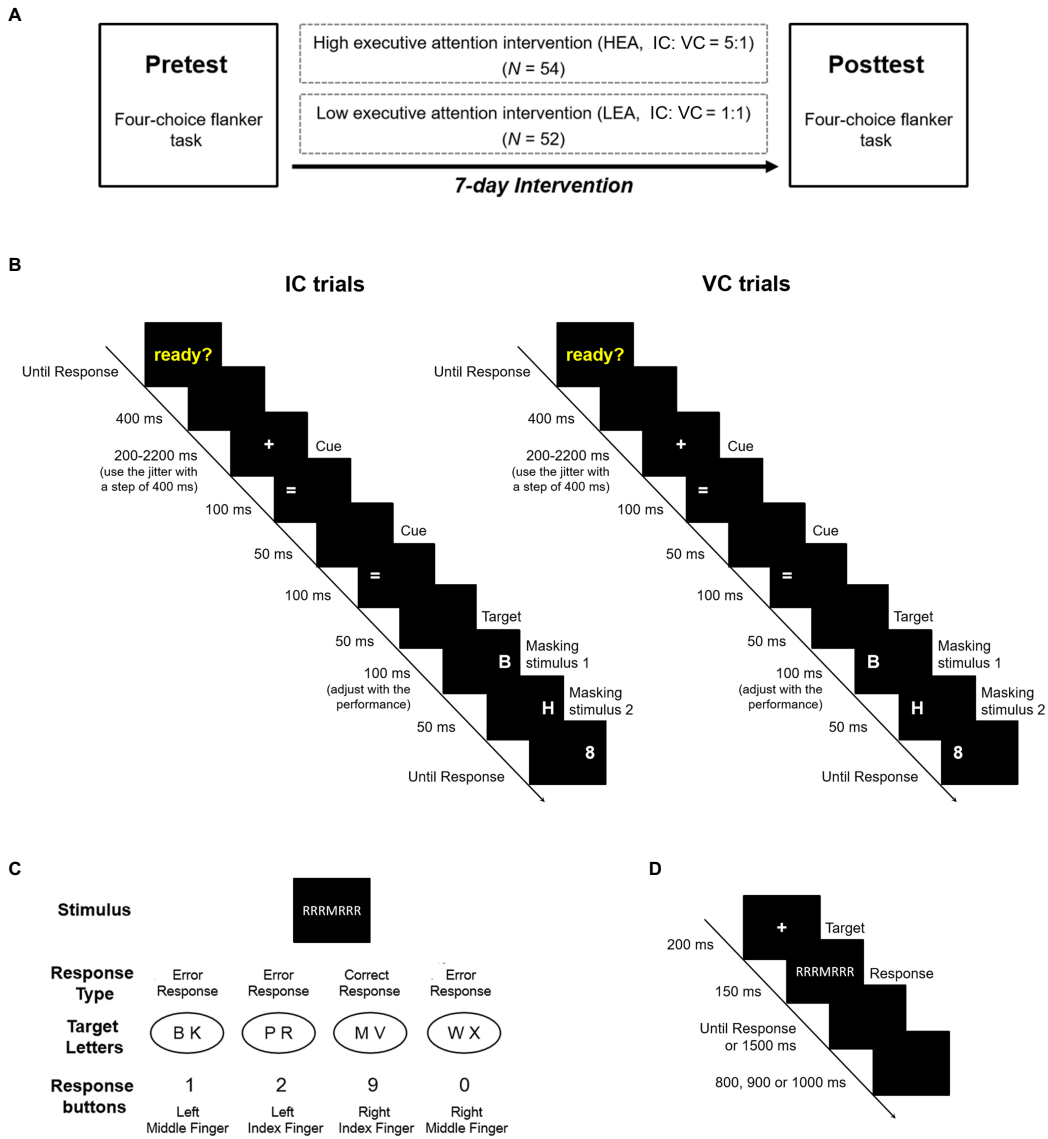
**(A)** Experimental design. **(B)** The Posner cueing paradigm. The sequence and time course of invalid cue (IC) and valid cue (VC) trials in the task. **(C)** Stimulus–response mapping in the four-choice flanker task. Each of the four response buttons corresponded to two target letters. In the sample shown, if a response was provided with the right index finger, it would be classified as a correct response. If a response was provided with the remaining fingers, it would be classified as an error response. **(D)** The four-choice flanker task. The sequence and time course of one typical trial in the task.

We implemented a computerized intervention program and a pretest and posttest design (Figure 1A). Before the intervention, all participants performed the pretest in the four-choice flanker task, following which both groups had a seven-day continuous intervention session. During the intervention, the HEA group performed the Posner cueing paradigm with an IC: VC ratio of 5:1, while the LEA group completed the Posner cueing paradigm with an IC: VC ratio of 1:1. Seven days following the pretest, both groups participated in the posttest in the four-choice flanker task. In order to maintain motivation, both groups received similar instructions before each session, suggesting that participants were asked to grasp the stimulus–response rules and respond as quickly and accurately as possible (Benedetti et al., 2003; Long et al., 2019).

### Apparatus and tasks

The experiment was conducted using E-Prime software (Psychology Software Tools, Inc., Pittsburgh, PA, United States) and ran on a 17-inch Dell monitor with a refresh rate of 85 Hz and a resolution of 1,024 × 768. Participants sat approximately 60 cm away from the screen in a comfortable chair in a quiet laboratory, and the stimuli were presented on the central screen.

#### Posner cueing paradigm

The task began with the presentation of the yellow word “ready?” on the center of the screen. The word did not disappear until participants were ready and pressed the spacebar on the keyboard. In the task, each trial started with the appearance of the cue, followed by the presentation of the target. The cue and the target randomly appeared on the left or right position of the screen, and their positions were at times consistent (valid cue condition, VC) or inconsistent (invalid cue condition, IC). It was noteworthy that the ratio of IC to VC trials was 5:1 in the HEA group, but 1:1 in the LEA group. The targets were the capital letters “B” “P” and “R” corresponding to the response buttons “1” “2” and “3” (right index finger, right middle finger and right ring finger). Participants were asked to respond to the target as quickly and accurately as possible. For each trial, a blank screen with a duration of 400 ms was first presented, followed by the fixation point for 200–2,200 ms (using the jitter with a step of 400 ms). Then, two identical cues “=” were appeared continuously in the same position at an interval of 50 ms, and each cue was presented for 100 ms. After a 50 ms blank screen, the target stimulus for 100 ms was appeared in the same (VC trials) or opposite (IC trials) position of the cue (Figure 1B). A 50 ms masking stimulus “H” was presented in the identical position, followed by another masking stimulus “8”; the “8” continued to appear until a response was given. During the intervention, task difficulty adjusted dynamically with performance by manipulating the target duration, so as to ensure the adaptive intervention. At the beginning of the intervention, the target duration was 100 ms. Once the average accuracy rate exceeded 60% in a block, the target duration would be shortened by 10 ms in the next block; if not, the duration would be increased by 10 ms. However, the duration would not be less than 30 ms in order to maintain stimulus visibility. The two groups completed five intervention sessions, with each intervention session consisting of 144 trials (720 trials in total); this lasted approximately 30 min each day.

#### Four-choice flanker task

The stimulus consisted of eight letters (B, K, P, R, M, V, W and X) and six neutral symbols (§, $, %, &, # and ?). A total of 48 incongruent stimuli and 48 neutral stimuli were constructed with letters and neutral symbols. Participants were asked to respond to the central target letter and ignore the flankers on both sides, and to press “1” with the left middle finger, “2” with the left index finger, “9” with the right index finger and “0” with the right middle finger (Figure 1C). Each trial began with the appearance of the fixation point for 200 ms, followed by the presentation of the stimulus array for 150 ms (Figure 1D). Participants were instructed to respond to the target letter as quickly and accurately as possible during the response screen over the course of 1,500 ms. Once a response was given, the next trial started after a response–stimulus interval of 800, 900 or 1,000 ms. The experiment included eight blocks consisting of 96 trials each (for a total of 768 trials), which took approximately 40 min to complete.

### Statistical analysis

Statistical analyses were implemented in SPSS software (version 21.0; IBM, Armonk, NY, USA). For all statistical tests in this study, the alpha level was set to 0.05 and the effect size was consulted with regard to the partial eta-square values. The outliers, defined as values that were more than three standard deviations away from the individual mean, were removed from the analyses.

#### Intervention performance changes

For the HEA and LEA groups, we evaluated the intervention performance for each participant in each intervention session and computed the average accuracy of IC and VC trials during each intervention session. We adopted the paired samples *t*-tests to assess the performance differences from the first to the last intervention session.

#### Post-error performance

PES was calculated by the reaction time (RT) of correct trials following errors (EC) minus the RT of correct trials following correct responses (CC; RT_EC_ − RT_CC_) in the four-choice flanker task. PEA was defined as the difference between accuracy following errors and accuracy following correct responses (Rabbitt, 1966; Wang et al., 2015; Li et al., 2020) in the four-choice flanker task. The RT and accuracy were analyzed using an analysis of covariance (ANCOVA) with the Response type on trial *n*-1 (correct, error) and Time (pretest, posttest) as within-subject factors, Group (HEA, LEA) as a between-subject factor, and mean RT or mean accuracy at pretest as covariates.

#### Flanker effect

In order to examine the overall performance in the four-choice flanker task, the RT and accuracy were analyzed by adopting a repeated measures analysis of variance (ANOVA), with Time (pretest, posttest) as a within-subject factor, Group (HEA, LEA) as a between-subject factor. In addition, to investigate the effects of the intervention on the flanker effect, we used an ANCOVA with Congruency (incongruent, neutral) and Time (pretest, posttest) as within-subject factors, Group (HEA, LEA) as a between-subject factor, and mean RT or mean accuracy at pretest as covariates.

#### Generalized linear mixed-effects analysis

To explore whether the relative changes in accuracy of the IC and VC trials from pretest to posttest (Post-Pre IC accuracy, Post-Pre VC accuracy) could predict relative changes in PEA from pretest to posttest (Post-Pre PEA), generalized linear mixed-effects analyses were conducted using R statistical software, version 3.6.3 (R Core Team, 2016, Vienna, Austria), including the lme4 package, version 1.1-21 (Bates et al., 2015) and the lmerTest package, version 2.0-32 (Kuznetsova et al., 2016).

Prior to performing each analysis, models were constructed with continuous variables (Post-Pre IC accuracy, Post-Pre VC accuracy, Post-Pre PEA) which were centered and scaled to have a mean of 0 and an standard deviation (*SD*) of 1 across the data set, and the category variables (Group) which was entered using the sum and contrast. The continuous variables were fitted *via* a linear mixed-effects analysis adopting the lmer function, with restricted maximum likelihood estimation. The categorical variables were fitted *via* generalized linear mixed-effects models using the glmer function, with a logit link with maximum likelihood estimation.

In each model, the effects of interest and its interactions (plus the intercept) were defined as fixed effects, and the variation in the within-subjects intercept was defined as a random effect. The statistical significance of each fixed effect was analyzed *via* lmertest (Kuznetsova et al., 2016), with Satterthwaite’s approximation to the denominator degrees of freedom. The following formula defined the mixed effect model in the current analysis:


Y=Xβ+Zγ+ε

Here, *Y* indicates the response variable, *X* is the fixed effect design matrix, *β* represents the fixed effect coefficient, *Z* indicates the random effect design matrix, *γ* is the random effect coefficient, and *ε* represents the error term.

Adopting the syntax of the R package lme4, we constructed the mixed effect model as follows:


$$\begin{array}{l} \mathrm{lmer (dependent}\_var\sim 1+\left(\mathrm{fixed}\_\mathrm{effect}\_1^{*}\;\mathrm{fixed}\_\mathrm{effect}\_2\right)\\ +\;\left(1\;|\;\mathrm{Participant}\right)) \end{array}$$

This syntax indicates a model with a fixed effect on the overall model intercept (the initial ‘1’), fixed effects on all independent variables of interest and their interactions, and a random effect on the variation in intercept per participant (‘1 | Participant’).

Two separate models were constructed to investigate the effect of Post-Pre IC accuracy and Group (HEA vs. LEA) on Post-Pre PEA, and the effect of Post-Pre VC accuracy and Group (HEA vs. LEA) on Post-Pre PEA, respectively.

#### Correlation analysis

To explore the relationship between the accuracy of IC or VC trials and the PEA in the HEA group, we conducted a series of correlation analyses regarding the accuracy of IC trials and PEA, and the accuracy of VC trials and PEA, at pretest and posttest respectively. Further, we used correlation analyses on Post-Pre IC accuracy and Post-Pre PEA as well as Post-Pre VC accuracy and Post-Pre PEA. A stepwise regression analysis was performed with Post-Pre PEA as the dependent variable, and Post-Pre IC accuracy and Post-Pre VC accuracy as predictive factors.

#### Support vector machine classification

Based on the behavioral changes between the two groups, we classified the groups into an HEA group and a LEA group according to PEA. The changes in PEA were regarded as features to distinguish the two groups, and the 1,000-times permutation test was performed to verify the reliability of the classifications.

## Results

### Intervention performance changes

**Figure 2:**
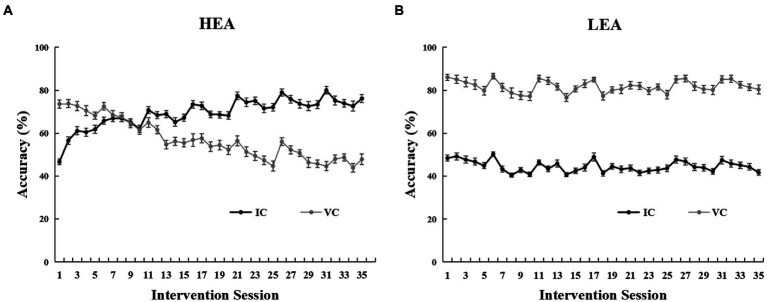
Intervention performance for the high executive attention (HEA) group **(A)**, and the low executive attention (LEA) group **(B)**, on the Posner cueing paradigm across intervention sessions. Error bars denote standard errors of the mean (*SEM*).

The intervention performance curves of the two groups are shown in Figure 2. For the HEA group, the accuracy of IC trials increased significantly from the first to the last intervention session (*t*(53) = −15.17, *p* < 0.001), improving by 29.46% (*SD* = 14.27%) on average; however, the accuracy of VC trials decreased significantly from the first to the last intervention session (*t*(53) = 7.64, *p* < 0.001), decreasing by 25.70% (*SD* = 24.72%) on average (Figure 2A; Table 1). For the LEA group, the accuracy of IC trials decreased significantly from the first to the last intervention session (*t*(51) = 4.23, *p* < 0.001), decreasing by 6.69% (*SD* = 11.42%) on average; the accuracy of the VC trials decreased significantly from the first to the last intervention session (*t*(51) = 2.50, *p* = 0.016), decreasing by 5.60% (*SD* = 16.16%) on average (Figure 2B).

**Table 1:** Descriptive statistics.

Measure	HEA group				LEA group			
	Pretest		Posttest		Pretest		Posttest	
	Mean	*SD*	Mean	*SD*	Mean	*SD*	Mean	*SD*
IC ACC (%)	46.69	9.36	76.15	14.19	48.40	10.37	41.71	9.50
VC ACC (%)	73.57	13.94	47.87	17.84	86.04	10.45	80.44	15.18
RT on CC trials	707.82	113.25	608.31	108.73	683.05	121.04	588.77	110.32
RT on EC trials	739.83	128.13	630.70	115.96	729.97	152.91	614.47	136.05
Post-correct ACC (%)	92.50	4.73	93.08	6.03	91.07	10.33	93.14	8.32
Post-error ACC (%)	90.34	6.92	93.17	6.27	89.51	11.31	89.52	13.93
RT in the flanker task	715.10	114.67	613.39	110.80	689.32	125.57	592.64	112.90
ACC in the flanker task (%)	92.04	4.80	93.60	5.40	90.88	10.24	92.30	10.20
RT on incongruent trials	719.35	116.22	617.76	109.68	697.92	131.01	598.00	107.49
RT on neutral trials	695.36	114.21	590.89	111.94	669.84	122.03	574.67	105.59
ACC on incongruent trials (%)	91.99	4.81	93.69	5.31	90.86	10.21	92.39	9.94
ACC on neutral trials (%)	92.24	4.80	94.03	5.32	91.02	10.30	92.95	9.21

ACC, accuracy; RT, reaction time; CC, correct trials following correct responses; EC, correct trials following errors; SD, standard deviation.

### Effects of intervention on the post-error performance

**Figure 3:**
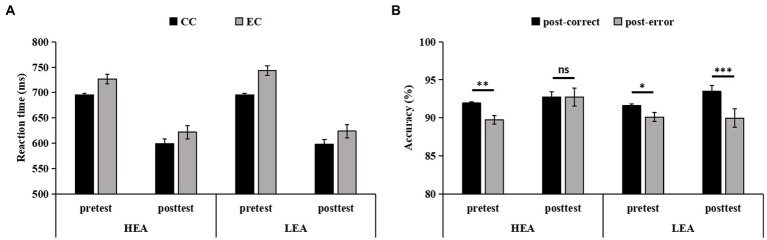
The reaction time (RT) **(A)**, and accuracy **(B)**, on trials following correct responses and errors in the four-choice flanker task for the high executive attention (HEA) and the low executive attention (LEA) groups at pretest and posttest. Error bars denote standard errors of the mean (*SEM*).

The Response type on trial *n*-1 × Time × Group ANCOVA of the RT in the four-choice flanker task revealed the main effect of Time (*F*(1,103) = 4.18, *p* = 0.043, = 0.04), indicating that the RT was significantly faster at posttest than at pretest. Other main effects (*ps* > 0.582) or interactions (*p*s > 0.349) were not significant, showing that there were no differences in RT on correct trials following errors and correct responses from pretest to posttest between the two groups (Figure 3A).

The Response type on trial *n*-1 × Time × Group ANCOVA of the accuracy in the four-choice flanker task showed main effects of Response type on trial *n*-1 (*F*(1,103) = 4.97, *p* = 0.028,  = 0.05) and Time (*F*(1,103) = 17.51, *p* < 0.001,  = 0.15), indicating that the accuracy on trials following errors was significantly lower than on trials following correct responses, and that the accuracy was significantly higher at posttest than at pretest. But the main effect of Group (*p* = 0.509) and all two-way interactions (*p*s > 0.095) did not reach significance. The three-way interaction was significant (*F*(1,103) = 9.78, *p* = 0.002,  = 0.09). *Post hoc* tests showed that, for the HEA group, the accuracy on trials following errors was significantly lower than on trials following correct responses at pretest (*F*(1,103) = 10.05, *p* = 0.002,  = 0.09); of note, the accuracy did not differ between the trials following errors and the trials following correct responses at posttest (*F*(1,103) = 0.01, *p* = 0.992). For the LEA group, the accuracy on trials following errors was significantly lower than on trials following correct responses at pretest (*F*(1,103) = 4.74, *p* = 0.032,  = 0.04) and at posttest (*F*(1,103) = 17.68, *p* < 0.001,  = 0.15; Figure 3B).

### Effects of intervention on the flanker effect

The Time × Group ANOVA of the RT in the four-choice flanker task revealed a main effect of Time (*F*(1,104) = 166.57, *p* < 0.001,  = 0.62), showing that the mean RT for both groups was faster at posttest than at pretest. The main effect of Group (*p* = 0.275) as well as the interaction (*p* = 0.744) were not significant.

The Time × Group ANOVA of the accuracy in the four-choice flanker task showed a main effect of Time (*F*(1,104) = 6.00, *p* = 0.016,  = 0.05), indicating that the mean accuracy for both groups was higher at posttest than at pretest. The main effect of Group (*p* = 0.396) as well as the interaction (*p* = 0.907) did not reach significance.

**Figure 4:**
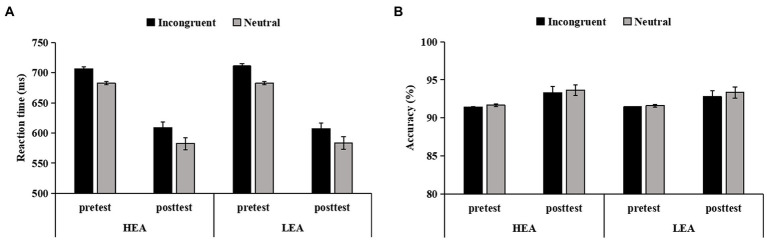
The reaction time (RT) **(A)**, and accuracy **(B)**, on incongruent and neutral trials in the four-choice flanker task for the high executive attention (HEA) and the low executive attention (LEA) groups at pretest and posttest. Error bars denote standard errors of the mean (*SEM*).

The Congruency × Time × Group ANCOVA of the RT in the four-choice flanker task revealed the main effect of Time (*F*(1,103) = 7.60, *p* = 0.007, = 0.07), indicating that the RT was significantly smaller at posttest than at pretest. There was no significant other main effects (*ps* > 0.796) or interactions (*p*s > 0.202; Figure 4A).

The Congruency × Time × Group ANCOVA of the accuracy in the four-choice flanker task showed the main effect of Time (*F*(1,103) = 22.69, *p* < 0.001,  = 0.18), suggesting that the accuracy was significantly higher at posttest than at pretest. Other main effects (*ps* > 0.680) or interactions (*p*s > 0.441) did not reach significance (Figure 4B).

### Generalized linear mixed-effects analysis results

The linear mixed-effects model of Post-Pre IC accuracy and Group on Post-Pre PEA showed that neither the main effects nor the interaction between Post-Pre IC accuracy and Group was significant (*ps* > 0.082).

**Figure 5:**
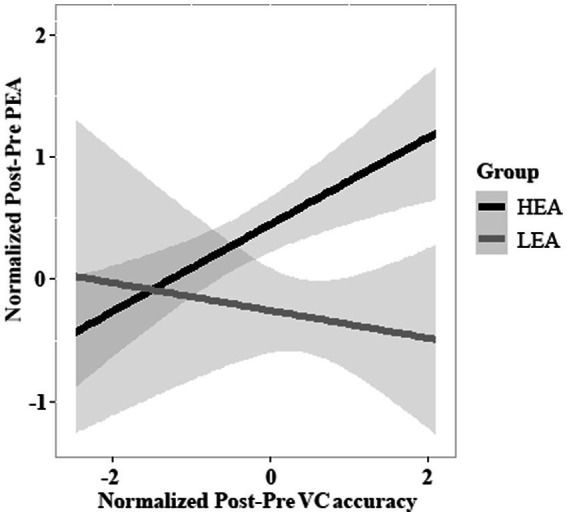
The relations between Post-Pre VC accuracy and Post-Pre PEA. This figure depicts the predicted values for the Post-Pre PEA, relative to Post-Pre VC accuracy and Group (high executive attention [HEA] vs. low executive attention [LEA]). The Post-Pre VC accuracy and Group interact, such that the influence of Post-Pre VC accuracy on Post-Pre PEA differs between the HEA and LEA groups. The shaded region around each line denotes standard errors of the mean (*SEM*).

The model of Post-Pre VC accuracy and Group on Post-Pre PEA revealed an effect of Post-Pre VC accuracy (estimate = 0.827, *SE* = 0.303, *df* = 102, *t* = 2.733, *p* = 0.007), suggesting that a greater Post-Pre VC accuracy was associated with a larger Post-Pre PEA, as well as an effect of Group (estimate = −0.700, *SE* = 0.204, *df* = 102, *t* = −3.427, *p* < 0.001), indicating that Post-Pre PEA was greater in the HEA group than in the LEA group. Crucially, there was a significant interaction in which Post-Pre VC accuracy predicted Post-Pre PEA as a function of Group (estimate = −0.470, *SE* = 0.221, *df* = 102, *t* = −2.127, *p* = 0.036; Figure 5). The interaction implied that increased Post-Pre VC accuracy predicted improved Post-Pre PEA in the HEA group, but not in the LEA group.

### Correlation analysis results

**Figure 6:**
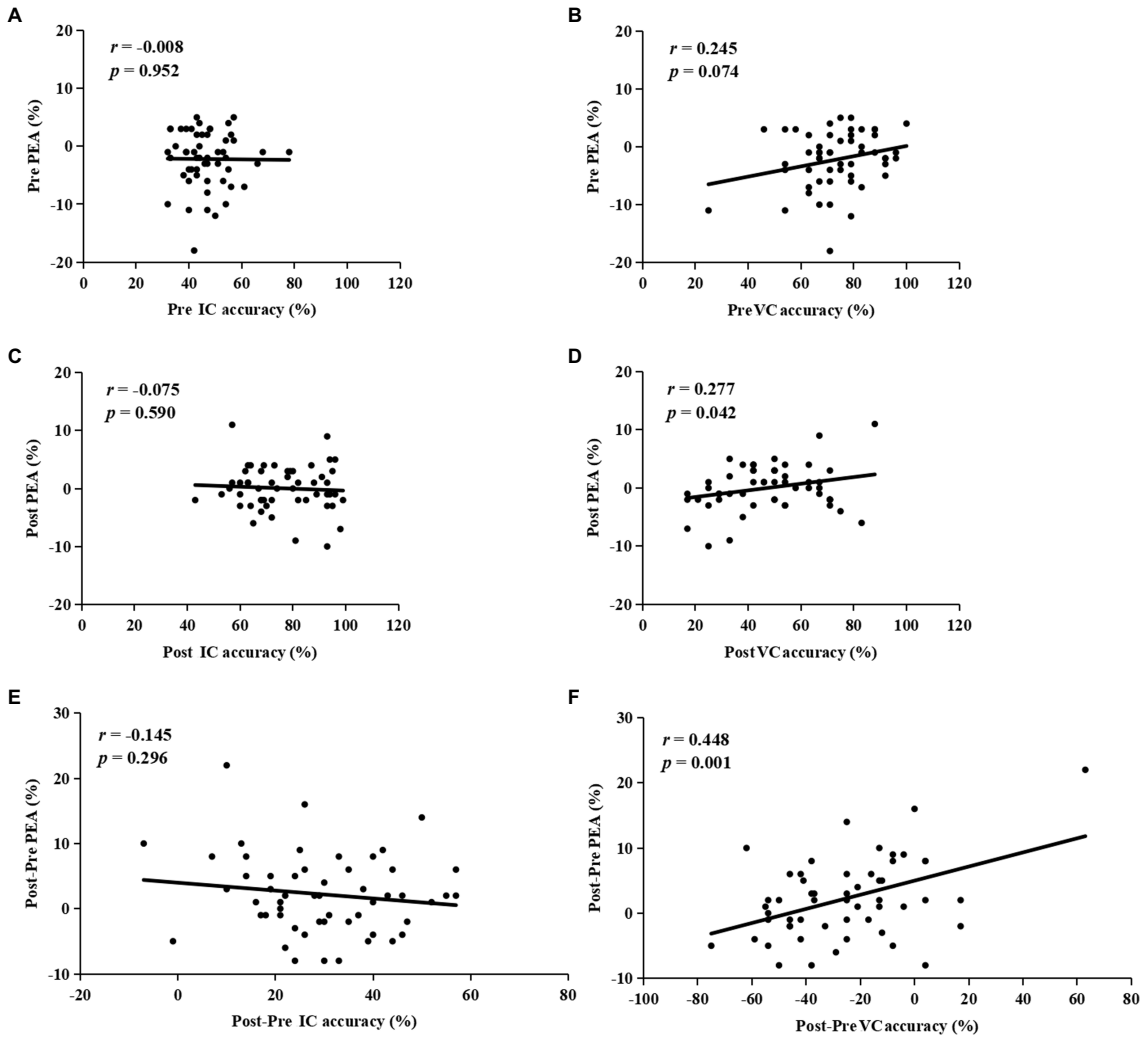
The correlations between the accuracy of invalid cue (IC) or valid cue (VC) trials and the post-error accuracy (PEA) at pretest **(A,B)**, and posttest **(C,D)**. The correlations between Post-Pre IC accuracy **(E)**, or Post-Pre VC accuracy **(F)**, and Post-Pre PEA.

The correlation analysis results for the HEA group showed that at pretest, there was no correlation with the accuracy of IC (*r* = −0.008, *p* = 0.952) or VC (*r* = 0.245, *p* = 0.074) trials and the PEA (Figures 6A,B); at posttest, a significant positive correlation was found between the accuracy of VC trials (*r* = 0.277, *p* = 0.042) and the PEA, but this result was not found in IC trials (*r* = −0.075, *p* = 0.590; Figures 6C,D). Notably, a significant positive correlation was observed between Post-Pre VC accuracy (*r* = 0.448, *p* = 0.001) and Post-Pre PEA, but not for Post-Pre IC accuracy (*r* = −0.145, *p* = 0.296; Figures 6E,F). Further, the stepwise regression analysis results revealed that Post-Pre VC accuracy was the only variable that could enter the regression model, which accounted for 20.1% variance of the changes in PEA from pretest to posttest (*F*(1,53) = 13.09, *p* = 0.001).

### Support vector machine classification results

The pretest to posttest changes in the PEA were regarded as a predictive variable, and the dichotomous variable (i.e., HEA and LEA groups) was defined as an outcome variable. The trained model for classifying the two groups reached 61.3% accuracy (permutation test: *p* = 0.007).

## Discussion

With the LEA group as a contrast, this study investigated whether the improvement induced by the EA intervention transferred to post-error performance. Summarizing the main results, the seven-day intervention on the Posner cueing paradigm showed an improvement in the accuracy of IC trials for the HEA group along with a reduction in the accuracy of IC trials for the LEA group after intervention, indicating that a high ratio of the Posner cueing paradigm intervention significantly improved the EA. Importantly, for the HEA group, the accuracy on trials following errors was lower than on trials following correct responses at pretest, but did not differ at posttest. Instead, for the LEA group, the accuracy on trials following errors was lower than on trials following correct responses at both pretest and posttest. This indicated the far transfer of the EA intervention to post-error performance. After intervention, the impaired post-error performance was recovered in the HEA group, but not in the LEA group. In addition, the PES did not differ between the two groups at pretest and posttest, suggesting that the transfer effect of the EA intervention was only reflected in the improvement in the PEA. It was noteworthy that for the HEA group, the changes in the accuracy of VC trials positively predicted changes in the PEA, and there was also a significant positive correlation between them. The former explained 20.1% variance of the latter. Moreover, the discrimination model constructed by machine learning had an acceptable prediction effect for the HEA group and the LEA group, which reached 61.3% accuracy. However, the flanker effect was not regulated by the EA intervention.

Notably, compared with the pretest, the accuracy of IC trials was improved, but the accuracy of VC trials was decreased at posttest in the HEA group. At the early stages of intervention, the processing of IC trials was active, which needs to “actively catch” attention resources to prevent automatic eye movements and induce endogenous correct eye movements (Unsworth et al., 2011). In contrast, the processing of VC trials was reflexive, in that eye movement tasks are performed automatically (Draheim et al., 2021). However, at the late stages of intervention, participants in the HEA group have learned the relative ratio of IC and VC trials in the Posner cueing paradigm. The processing efficiency of IC trials was improved due to continuous practice. Therefore, participants have expectations for the IC trials and form a habitual mode of responding to the opposite position of the cue. In other words, they have learned that the target would appear in the opposite position of the cue in a high ratio. Thus, the strategy to enhance performance is to immediately transfer attention to the opposite position of the cue once the cue is monitored. At this stage, the processing of IC trials is reflexive, in that the task is completed automatically. The attention shift path should be that attention is shifted from the same position to the opposite position of the cue (same-opposite). However, the processing of VC trials is active, which needs to “actively catch” attention resources. In the attention shift path, attention is shifted from the same position to the opposite position of the cue, and then back to the same position of the cue (same-opposite-same). So the VC trials are more difficult than the IC trials, and actively catch more EA. This way, the EA intervention reversed the EA processing for different types of trials. Of note, the reversal did not mean that the processing of VC trials at posttest was equivalent to that of IC trials at pretest; because there was a correlation between the accuracy of VC trials and the PEA at posttest, but not between the accuracy of IC trials and the PEA at pretest. This prompted that, at posttest, the accuracy of VC trials became an effective index to measure EA, reflecting the ability to “actively catch” attention resources. For the HEA group, the higher the accuracy in VC trials at posttest, the better the EA.

The present study confirmed that EA intervention with a high ratio of Posner cueing paradigm effectively improved the PEA. This prompts the question of how the transfer effect from EA intervention to PEA came into being. Based on the result that the changes in the accuracy of VC trials predicted changes in the PEA, we analyzed the underlying causes of the transfer effect from the characteristics of VC trials and their associated processing mechanisms.

First, from the characteristics of VC trials, VC trials can be regarded as deviant (i.e., with a presentation probability less than 30%) or novel stimuli (Näätänen, 1990), when the ratio of IC and VC trials is 5:1. Importantly, previous studies pointed out that the deviant or novel stimuli would involuntarily capture attention (Parmentier et al., 2008; Pacheco-Unguetti and Parmentier, 2014). Yet, as the intervention went on, EA was improved continuously and voluntary/active attention control was enhanced. Meanwhile, VC trials continued to be trained repeatedly. At the late stages of intervention, the processing mechanism of VC trials may reverse, switching from involuntarily to voluntarily catch more attention resources, so as to better suppress bottom-up interference and conduct top-down attention control, thus improving the performance of VC trials. That is, EA intervention improved the ability to “actively catch” more attention resources in the VC trials (i.e., deviant or novel stimuli). Similarly, error is also a deviant or novel event, resulting in shared processing with the VC trials. Thus, the ability to “actively catch” more attention resources in the VC trials is applicable in the post-error flanker task and optimizes post-error performance.

Second, from the processing mechanism of the VC trials, participants first need to match the attention location with the target location when VC trials are presented. Under the condition that the ratio of IC and VC trials is 5:1, VC trials as the deviant or novel stimuli mainly complete two stages of processing: one is to deal with the mismatch between the attention on the opposite position of the cue and the target on the same position of the cue; one is to adjust attention to make a goal-oriented response for the VC trials (Menon and Uddin, 2010). Notably, due to the novelty of VC trials, the processing in the first stage would occupy a lot of attention resources, resulting in little attention resources being left for the second stage, which impairs the performance of VC trials. With intervention, the mismatch processing would gradually become automated, so that the processing in the first stage would occupy fewer attention resources, leaving more attention resources for the second stage and finally improving the performance of VC trials. It is worth noting that when errors occur in the four-choice flanker task, participants encounter similar processing: one is to solve the mismatch between errors and correct responses in the previous trial; the other is to adjust attention to complete the current trial. Moreover, the studies pointed out that the error-related processing from the previous trial (error monitoring) occupied more central resources, while fewer attention resources were available for the current trial (post-error adjustments; Jentzsch and Dudschig, 2009; Buzzell et al., 2017; Li et al., 2021). The benefits of automatically solving mismatch processing obtained in the EA intervention are applicable in the post-error flanker task. After intervention, once errors occur, mismatch processing is automated and employs fewer attention resources, leaving more attention resources to complete the post-error adjustments for the current trial; this is conducive to the improvement in post-error performance.

It is worth noting that the PES effect was not regulated by the EA intervention in our study. According to adaptive theories of error processing, errors trigger a cascade of processes that represents a remedial effort of the cognitive system, with a clear goal of avoiding future errors (Laming, 1968; Ridderinkhof, 2002; King et al., 2010; Danielmeier et al., 2011). Thus, improving behavioral accuracy after errors is the ultimate goal of error processing. Among them, the most representative conflict monitoring theory proposes that errors induce a more conservative speed-accuracy trade-off strategy, namely improving accuracy after errors by slowing response speed (Botvinick et al., 2001; Cavanagh et al., 2011; Purcell and Kiani, 2016). Based on the above proposals, after obtaining intervention benefits, participants may give priority to ensure the accuracy after errors rather than speed in the current study. Moreover, the current results showed that the EA intervention benefits can only help PEA restore to the same level as the post-correct accuracy. Nevertheless, if greater intervention benefits obtained by increasing the session or the difficulty of the EA intervention transfer to error processing, resulting in a high enough PEA, the remaining intervention benefits are very likely to act on the response speed improvements after errors.

However, our results demonstrated that the intervention gains on the Posner cueing paradigm also did not impact the flanker effect. Firstly, considering that the ratio of incongruent to neutral trials is 1:1 in the four-choice flanker task, there are no deviant or novel stimuli. Thus, after the EA intervention, participants cannot “actively catch” more attention resources for conflict processing. Secondly, the conflict in the four-choice flanker task is a perceptual conflict induced by incongruent stimuli. This conflict processing only needs to inhibit the interference letters on both sides and respond to the central target letter (Verbruggen et al., 2006; Shu et al., 2019), which is an entirely different mechanism from the mismatch processing in the VC trials. Thus, the benefits on the mismatch processing obtained in the EA intervention could not be transferred to the conflict processing. Collectively, there is no shared processing between the Posner cueing paradigm intervention and the conflict processing, thereby offering an understanding as to why the EA intervention did not improve the flanker effect.

Additionally, there were subtle differences in overall response accuracy across task and across conditions within tasks in the present study. According to Steinborn et al. (2012), these subtle differences yield substantial differences in post-error performance, in which post-error adjustments are larger when overall accuracy is high but smaller when overall accuracy is low. We discussed the effect of this limitation on the interpretation of the current results as follows. First, these findings by Steinborn et al. (2012) are observed in the self-paced task (i.e., response–stimulus interval was zero). Burns (1971) argues that error-induced orienting response decays over time (e.g., with long response–stimulus intervals). Moreover, Jentzsch and Dudschig (2009) point out that a post-error refractory period is about 200–300 ms. In the present study, the response–stimulus interval was long (more than 800 ms), so post-error performance was less affected by the subtle differences in overall accuracy. Second, even if the influence of subtle differences did exist, it did not seem to disturb our interpretation of the results. Based on Steinborn et al. (2012), the higher accuracy participants performed overall, the stronger post-error accuracy decrease they responded. In the present study, both the HEA and LEA groups worked post-error accuracy decrease at pretest. Compared with the pretest, the overall accuracy was increased at posttest in both groups. But at posttest, there was no post-error accuracy decrease in the HEA group, the post-error accuracy decrease was greater in the LEA group. This indicates that the intervention gains on the Posner cueing paradigm is first used to counteract the interferences caused by the high overall accuracy, and then to improve the accuracy following errors, demonstrating that EA intervention does improve post-error performance. Third, unlike the task instruction focuses on accuracy (Jentzsch and Leuthold, 2006), we put emphasis on speed and accuracy with equal weight, which reduces the impact of emphasizing high accuracy on the results to a certain extent. Therefore, the subtle differences in overall accuracy across conditions do not affect the main interpretation of our results. Future research should consider the trait variables related to the overall accuracy to more fully interpret these results.

In summary, the causal role of EA intervention in improving post-error performance is to promote the newly acquired capabilities of the “actively catch” more attention resources and the automatic mismatch processing. When an error occurs, the acquired capabilities help to actively catch more attention resources, and minimize the occupation of attention resources in the mismatch processing (error monitoring). The combination of the two capabilities effectively diminishes the central bottleneck stage induced by error monitoring (Jentzsch and Dudschig, 2009), and instead supplies more attention resources for the post-error adjustments. This is conducive for the target-related processing in the current trial, thereby improving the PEA. This means that when more attention resources are available for the post-error adjustments, the negative impacts of errors on accuracy in the current trial would disappear. The above results offer causal evidence for a deeper understanding of the mechanisms underlying post-error adjustments; however, empirical data for these capabilities is insufficient and requires further study to address this phenomenon. In general, the study provides a new approach and perspective for further understanding the causal mechanism of EA on error processing by verifying the effectiveness of an EA intervention.

## Data availability statement

The raw data supporting the conclusions of this article will be made available by the authors, without undue reservation.

## Ethics statement

The studies involving human participants were reviewed and approved by Southwest University Human Ethics Committee for the Human Research. The patients/participants provided their written informed consent to participate in this study.

## Author contributions

QL designed the tasks. YL and XW collected the data. QL and MZ analyzed the data. FS provided language help. QL and AC wrote the manuscript. QL, XC and AC revised the manuscript. All authors approved the final version of the manuscript for submission.

## Funding

This work was supported by grants from the National Natural Science Foundation of China (32171040, 31900803) and Chongqing Research and Innovation Funds for Postgraduates (CYB22096).

## Conflict of interest

The authors declare that the research was conducted in the absence of any commercial or financial relationships that could be construed as a potential conflict of interest.

## Publisher’s note

All claims expressed in this article are solely those of the authors and do not necessarily represent those of their affiliated organizations, or those of the publisher, the editors and the reviewers. Any product that may be evaluated in this article, or claim that may be made by its manufacturer, is not guaranteed or endorsed by the publisher.
